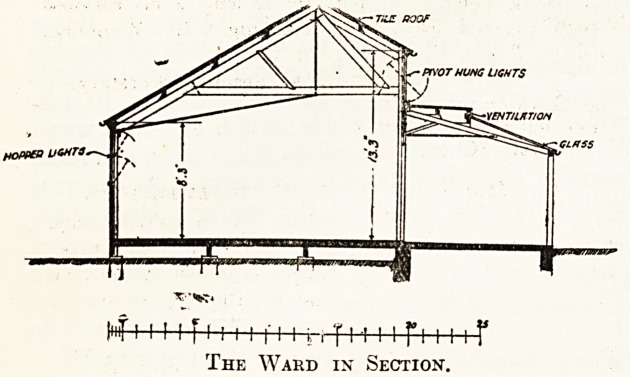# The New Soldiers' Wards at Norwich Hospital

**Published:** 1915-02-27

**Authors:** 


### THE NEW SOLDIERS' WARDS AT NORWICH HOSPITAL.

### A Suggestive Type for Continued Treatment.

Ever since the war commenced the Norfolk and Norwich Hospital have been taking in wounded soldiers at the rate of 100 every ten to fourteen days. These have been accommodated mostly in tents, but the committee found that a few soldiers in each convoy remained on their hands for continued treatment, and therefore a building of a more permanent nature (although temporary) was required. Appended are the plans and description of the building which, as recorded last week, has been erected, and was actually opened on February 11. These particulars are of interest, because the solution of the problem by the hospital staff at the Norfolk and Norwich Hospital will be useful to other institutions similarly situated.

The illustrations show in photographic view plan and sections of the emergency block in the grounds for the accommodation of wounded soldiers, which has been erected by public subscription, through the agency of the *Norfolk News* Company, Limited, and is named after the Eastern Daily Press.

The building has a total length of 222 feet; the front having a southern aspect. The centre portion is an administration block, consisting of a kitchen, larder, store, etc. The kitchen is provided with a projecting glass bay, commanding a view of either ward to facilitate service and supervision. Of course, the central administration block of the main hospital will be available to supply the varied wants of these wards.

The wards are placed on either side of the administration block, and have doorways at intervals giving easy access to a glazed verandah, 10 feet wide, which runs the whole length of either ward. Each ward is shown to contain thirty beds, and room remains for a few more if necessary. At the rear of the building is placed the sanitary block, consisting of bathrooms, sink room, w.c.'s and urinals, cut off from the wards by sheltered but open-air lobbies.

This number of beds in relation to cubical capacity is in excess of the usual hospital practice, but the management of the Norfolk and Norwich Hospital have in most cases adopted, apparently with great success, an open-air treatment, and except during excessively inclement weather a large number of these beds will be placed on the verandah.

A great deal of care has been expended on the design of the wards to obtain a thorough and ample system of natural ventilation. For this purpose the wall plate on the north wall is kept at a level of 8 feet 3 inches, whilst that on the south side is 13 feet 3 inches, allowing a series of pivot-hung windows above the verandah. The verandah is partly glass and partly flat, with ventilation

The Ground Plan of the New (Temporary) Soldiers' Wards.

General View of the Emergency Ward.

between the two to prevent the atmosphere under the glass becoming overheated during hot weather. The building is an effort to produce wards which, although capable of being shut in in bad weather,

The Ward in Section.

are really open-air wards. Every part of every door and window will open to the fullest extent.

Structure, Equipment and Cost.

The walls are framed in wood, covered both internally and externally with asbestos sheeting. The roof is tiled on boarding and the floor is of wood. The windows on the northern side, also those between the doors leading on to the verandah, open as hoppers, whilst the clerestory windows above the verandah are pivot-hung. The wards are heated with two hot-air stoves, and a hot-water supply has been installed to supply the service to the baths, sinks, etc. The whole building is lighted by electricity. No painting or colouring has been undertaken at the present time as immediate occupation is necessary. Cost was a prime consideration, and the ward was designed as plainly and inexpensively as possible, compatible with the object to be served, the total being £1,500, including lighting, hot-water supply, etc. The architects were Messrs. Edward Boardman and Son, and the builders, Messrs. J. Youngs and Son, Limited, both of Norwich.

This new building will bring the total accommodation for war patients up to 225, four tents having been previously erected. The Board is now enabled to provide for the care of soldiers requiring prolonged treatment, and they expect to comply with the desire of the War Office that they will receive a hundred fresh war patients every week. Out of the sixty beds fifty-six have now been endowed as a result of the appeal to employees of large firms to give £5 a month for their maintenance.

---

Caersws.—The Newtown and Llanidloes Guardians have decided to carry out alterations at the workhouse, and to instal a new heating system. The total cost is estimated at £2,000.

## Figures and Tables

**Figure f1:**
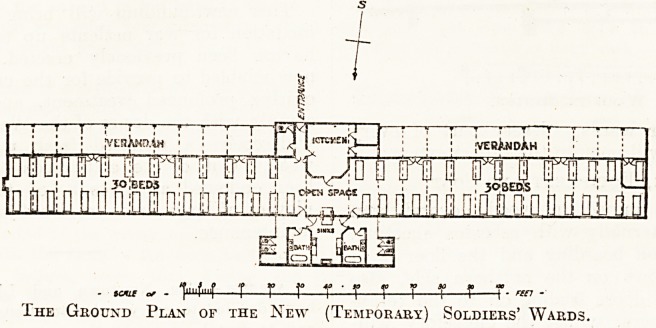


**Figure f2:**
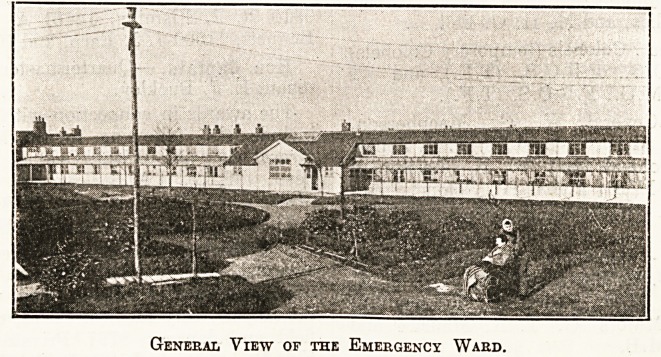


**Figure f3:**